# Procedural Optimization in CT-Guided Lung Biopsy: Impact of Needle Angle and Patient Positioning on Complication Rates

**DOI:** 10.3390/diagnostics16121792

**Published:** 2026-06-10

**Authors:** Erkan Bilgin, Ahmet Bayrak, Çetin İmamoğlu, Ezel Yaltırık Bilgin, Almıla Coşkun Bilge, Elif Aktaş, Hüseyin Çakmak, Banu İnce Alkan

**Affiliations:** 1 Department of Radiology, Dr. Abdurrahman Yurtaslan Ankara Oncology Training and Research Hospital, Ankara 06200, Türkiye; kaysbayrak@gmail.com (A.B.); cetinimamoglu@gmail.com (Ç.İ.); ezelyaltirik@yahoo.com (E.Y.B.); almilacoskun@gmail.com (A.C.B.); elifaktasmd@gmail.com (E.A.); 2 Department of Thoracic Surgery, Dr. Abdurrahman Yurtaslan Ankara Oncology Training and Research Hospital, Ankara 06200, Türkiye; cakmak1huseyin@hotmail.com; 3 Department of Pathology, Dr. Abdurrahman Yurtaslan Ankara Oncology Training and Research Hospital, Ankara 06200, Türkiye; banuincealkan@gmail.com

**Keywords:** CT-guided lung biopsy, pneumothorax, hemorrhage, procedural optimization, needle angle, patient positioning

## Abstract

**Background/Objectives:** CT-guided lung biopsy is an essential diagnostic procedure but is associated with complications such as pneumothorax and pulmonary hemorrhage. While patient- and lesion-related factors are well established, operator-modifiable parameters remain less clearly defined. This study aimed to evaluate complication rates and identify independent predictors, with a particular focus on modifiable procedural factors. **Methods:** This retrospective study included 309 consecutive patients undergoing CT-guided transthoracic lung biopsy. Patient, lesion, and procedural variables—including needle–pleura angle and patient positioning—were analyzed. Complications were classified according to the Society of Interventional Radiology (SIR) system. Multivariate logistic regression analyses were performed to identify independent predictors. **Results:** Complications occurred in 20.4% of patients, with pneumothorax (14.2%) and hemorrhage (4.9%) being the most frequent. A needle angle ≤ 65° was independently associated with major complications (OR = 8.12, *p* = 0.025). Perilesional emphysema (OR = 19.38, *p* = 0.004) and pleural effusion (OR = 30.72, *p* = 0.001) were also strong predictors. Supine positioning significantly increased hemorrhage risk (OR = 9.03, *p* = 0.043). **Conclusions:** Operator-modifiable factors, particularly needle angle and patient positioning, may influence complication risk. Optimization of these parameters may provide a practical approach to improving procedural safety in CT-guided lung biopsy.

## 1. Introduction

CT-guided lung biopsy is a widely used, minimally invasive procedure and remains a cornerstone in the diagnostic evaluation of pulmonary lesions, particularly when alternative methods such as transbronchial biopsies are not feasible or have failed [1]. While this technique is highly effective in obtaining tissue samples for histopathological analysis, it is not without risks. The most common complications associated with CT-guided lung biopsy include pneumothorax and pulmonary hemorrhage, which may range from self-limiting minor events to severe outcomes requiring hospitalization [2]. Understanding the frequency, severity, and risk factors of these complications is crucial for optimizing procedural safety and minimizing patient morbidity.

The reported incidence of pneumothorax following CT-guided lung biopsy varies widely, with rates ranging between 12% and 45%, while approximately 2–15% of cases require chest tube placement [3]. Pulmonary hemorrhage, on the other hand, has been documented in 4–27% of procedures, often presenting as perilesional or needle tract hemorrhage on post-biopsy imaging [4]. Although the majority of these complications are minor, severe cases can lead to prolonged hospital stays, increased healthcare costs, and delays in definitive treatment.

Several studies have investigated patient-, lesion-, and technique-related factors contributing to post-biopsy complications; however, most have focused predominantly on non-modifiable variables. Older age, presence of emphysema, small lesion size, increased lesion-to-pleura distance, multiple pleural passes, and steep needle trajectory have been frequently reported as significant risk factors for pneumothorax and hemorrhage [5,6,7]. Additionally, perilesional emphysema and pleural effusion have been identified as independent predictors of major complications, likely due to their effects on lung elasticity and hemostatic control [8]. However, variability in reported risk factors persists, highlighting the need for further investigation focusing on operator-modifiable procedural parameters and standardized classification systems such as the Society of Interventional Radiology (SIR) grading scale [9].

Therefore, this study aimed to evaluate the incidence and severity of complications using the SIR classification and to identify independent predictors, with a particular emphasis on operator-modifiable procedural factors that may offer practical opportunities for reducing complication risk.

## 2. Materials and Methods

### 2.1. Study Design and Population

This retrospective cohort study was conducted at a tertiary referral center and included 309 consecutive patients who underwent CT-guided lung biopsy between February 2023 and October 2024. The study was reported in accordance with the Strengthening the Reporting of Observational Studies in Epidemiology (STROBE) guidelines, and the completed STROBE checklist is provided in Supplementary Table S1. The study was approved by the Institutional Review Board (Approval No: 2024-107-158, approved on 31 October 2024). The requirement for informed consent was waived due to the retrospective nature of the study.

Clinical trial number: not applicable.

### 2.2. Inclusion Criteria

Patients were eligible for inclusion if they met the following criteria:

- Underwent CT-guided transthoracic lung biopsy for suspected pulmonary lesions.
- Had complete imaging and clinical records available, including pre-procedural chest CT, procedural details, and post-procedural follow-up imaging.
- Met the required coagulation profile criteria (platelet count ≥ 50,000/mm^3^, prothrombin time > 60%, and international normalized ratio (INR) ≤ 1.5).
- Were ≥18 years of age at the time of the procedure.

### 2.3. Exclusion Criteria

Patients were excluded if they met any of the following criteria:

- Incomplete medical records or missing procedural or post-procedural imaging.
- Uncorrectable coagulopathy (platelet count < 50,000/mm^3^ or INR > 1.5).
- Presence of an unbiopsiable lesion due to proximity to critical structures such as major blood vessels, mediastinum, or diaphragm.
- Patients with severe respiratory failure or an inability to tolerate the biopsy procedure.
- Non-cooperative patients leading to procedure cancellation.
- Patients with a prior biopsy of the same lesion within one month before the study period.

A flowchart summarizing patient selection and study inclusion is presented below:

Patients undergoing CT-guided transthoracic lung biopsy between February 2023 and October 2024
↓
Patients meeting inclusion criteria and having complete clinical and imaging data
↓
Final study cohort included in analysis (n = 309)

### 2.4. Biopsy Procedure

All biopsies were performed under CT guidance using a 16-slice multidetector CT scanner (GE Revolution, GE HealthCare, Milwaukee, WI, USA) by interventional radiologists with at least five years of experience. The biopsy pathway and patient positioning (supine or prone) were determined based on pre-biopsy CT imaging to provide the shortest and safest needle trajectory.

After local anesthesia with 1% lidocaine, a 20G coaxial or semi-automated core biopsy needle was used to obtain tissue samples. The number of pleural passes was minimized to reduce the risk of complications. Procedural CT acquisitions were obtained to confirm needle placement within the lesion. Post-procedure, immediate CT imaging was performed to assess complications such as pneumothorax or hemorrhage. Patients were monitored for at least 4 h, and a follow-up chest radiograph was taken to detect delayed complications.

### 2.5. Data Collection and Outcome Measures

**Figure 1:**
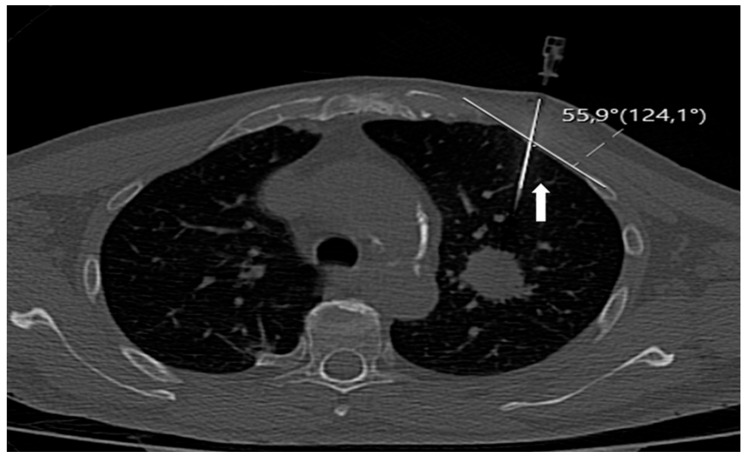
CT-guided transthoracic lung biopsy demonstrating measurement of the needle–pleura angle. The angle was measured between the biopsy needle trajectory and the pleural surface at the puncture site. The white arrow indicates the angle measurement, illustrating the reference lines drawn along the needle trajectory and pleural surface used to calculate the needle–pleura angle.

Patient demographics, lesion characteristics, procedural details, and complications were recorded. Collected data included:

- Patient factors: Age, gender, comorbidities, presence of emphysema.
- Lesion characteristics: Size, location, histopathology, distance from pleura, presence of perilesional emphysema or pleural effusion.
- Procedure-related factors: Biopsy position (supine/prone), needle angle, number of pleural passes, and duration of the procedure.
- The needle–pleura angle was defined as the angle formed between the biopsy needle trajectory and the pleural surface at the puncture site on CT images. Angles closer to 90° represented a more perpendicular needle approach to the pleura, whereas smaller angles indicated a more tangential trajectory (Figure 1).
- Complications: Pneumothorax, hemorrhage, and need for intervention (chest tube placement or blood transfusion).

### 2.6. Statistical Analysis

All statistical analyses were conducted using SPSS version 27 (IBM Corp., Armonk, NY, USA). Normality of continuous variables was assessed using the Kolmogorov–Smirnov test and graphical methods (Q-Q plot and histogram). Continuous variables were considered approximately normally distributed based on graphical assessment and normality testing; therefore, parametric statistical methods were applied.

Categorical variables were presented as frequency (%) and compared using the chi-square or Fisher’s exact test. Continuous variables were summarized as mean ± standard deviation (SD) and analyzed using one-way ANOVA or independent sample *t*-test.

To identify independent predictors of major complications, multinomial and binomial logistic regression models were constructed. The model’s goodness of fit was tested using the likelihood ratio test, and statistical significance was set at *p* < 0.05. Odds ratios (ORs) with 95% confidence intervals were reported.

## 3. Results

### 3.1. Patient and Lesion Characteristics

A total of 309 patients who underwent CT-guided transthoracic lung biopsy were included in the study. The mean age of the patients was 64.5 ± 11.6 years, with 186 (60.2%) being male and 123 (39.8%) female. The majority of biopsies (73.1%) were performed on malignant lesions, while 26.9% were benign.

The mean lesion diameter was 28.4 ± 15.2 mm, and the average lesion-to-pleura distance was 33.4 ± 21.3 mm. More than half of the lesions (53%) were located in the upper lobes (right or left). High-grade emphysema was present in 55 (17.8%) patients, while 22 (7.1%) had perilesional emphysema. Pleural effusion was noted in 17 (5.5%) cases, and 3 (1%) lesions involved a fissural transition.

Most biopsies (68%) were performed in the supine position, while 32% were done in the prone position. The majority of lesions (89%) had a solid morphology, while 3.9% were ground-glass opacity (GGO) and 7.1% were cavitary lesions. The detailed patient and lesion characteristics are summarized in Table 1.

### 3.2. Complications and Severity Classification

A total of 63 patients (20.4%) experienced complications. The most common complication was pneumothorax, occurring in 44 (14.2%) cases. Hemorrhage was observed in 15 (4.9%) patients, and 4 (1.3%) patients had both pneumothorax and hemorrhage.

According to the Society of Interventional Radiology (SIR) classification, the severity of complications was as follows:

- SIR 1 (minor, no intervention required): 49 (15.9%) patients.
- SIR 2 (minor, requiring short-term observation): 4 (1.3%) patients.
- SIR 3–4 (major, requiring intervention): 10 (3.2%) patients.

**Figure 2:**
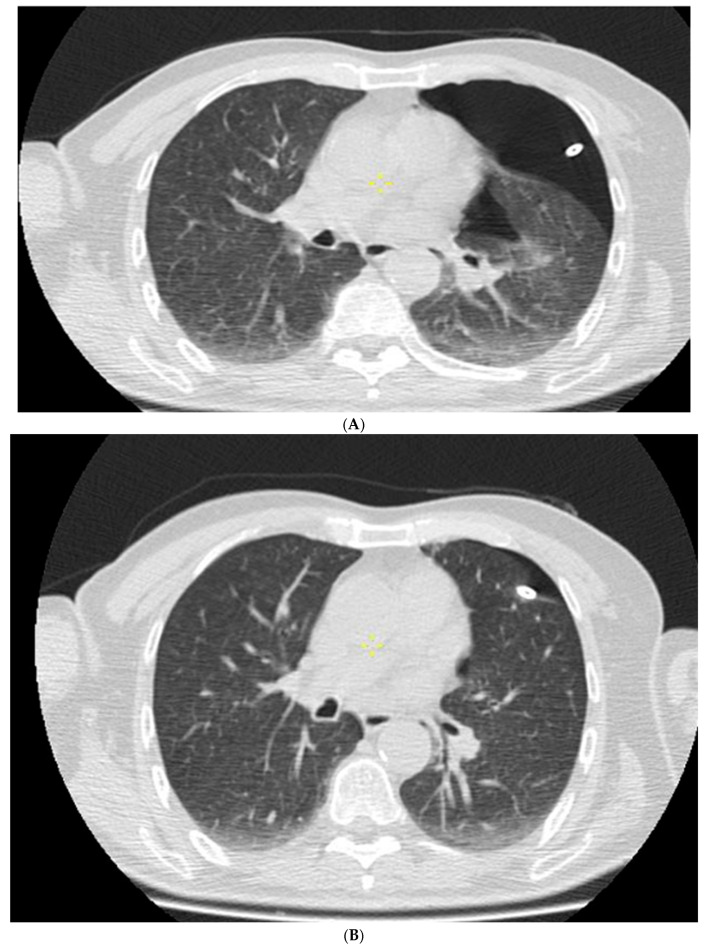
(**A**): Chest tube insertion after pneumothorax. (**B**): Resolution of pneumothorax after chest tube insertion.

Among patients with pneumothorax, 12 (27.3%) required chest tube placement, while others were managed conservatively (Figure 2). No patients experienced SIR 5–6 complications such as long-term sequelae or death.

### 3.3. Univariate Analysis of Risk Factors for Complications

#### 3.3.1. SIR Complication Severity

In the univariate analysis (Table 2), the following factors were significantly associated with higher SIR 2–4 complication rates:

- Older age (F = 3.719, *p* = 0.025);
- Larger lesion size (F = 5.325, *p* = 0.005);
- Increased lesion-to-pleura distance (F = 4.750, *p* = 0.009);
- Decreased skin-to-pleura distance (F = 8.607, *p* < 0.001);
- Presence of high-grade emphysema (χ^2^ = 38.299, *p* < 0.001);
- Presence of perilesional emphysema (χ^2^ = 26.733, *p* < 0.001);
- Presence of pleural effusion (χ^2^ = 22.568, *p* < 0.001);
- Needle angle ≤ 65° (χ^2^ = 9.995, *p* = 0.007).

**Table 2:** Comparison of SIR Classification According to Patient Characteristics (Univariate Analysis Results).

Continuous Variables	SIR0 (n = 246)	SIR1 (n = 49)	SIR2–4 (n = 14)	F	*p*-Value
Age (years), mean (±SD)	63.6 (12.1)	67.6 (8.7)	69.1 (9.4)	3.719 ^a^	0.025 ***
Lesion diameter (mm), mean (±SD)	28.7 (15.0)	24.3 (14.2)	39.0 (17.5)	5.325 ^a^	0.005 **
Skin–pleura distance (mm), mean (±SD)	41.4 (10.5)	39.3 (11.6)	29.4 (11.4)	8.607 ^a^	<0.001 *
Lesion–pleura distance (mm), mean (±SD)	31.6 (19.1)	39.2 (25.6)	44.6 (34.3)	4.750 ^a^	0.009 **
Ordinal and Nominal Variables	SIR0 (n = 246)	SIR1 (n = 49)	SIR2–4 (n = 14)	χ^2^	*p*-Value
Gender, n (%)				4.168 ^b^	0.124
Male	141 (75.8)	35 (18.8)	10 (5.4)		
Female	105 (85.4)	14 (11.4)	4 (3.3)		
Lesion location, n (%)				9.015 ^c^	0.466
Right upper	62 (84.9)	8 (11)	3 (4.1)		
Right lower	37 (78.7)	10 (21.3)	0 (0)		
Right middle	10 (66.7)	4 (26.7)	1 (6.7)		
Left upper	71 (78)	16 (17.6)	4 (4.4)		
Left lower	60 (78.9)	10 (13.2)	6 (7.9)		
Left lingular	6 (85.7)	1 (14.3)	0 (0)		
Lesion histopathology, n (%)				3.704 ^b^	0.157
Malign	174 (77)	41 (18.1)	11 (4.9)		
Benign	72 (86.7)	8 (9.6)	3 (3.6)		
Emphysema grade, n (%)				38.299 ^b^	<0.001 *
1–2	216 (85)	34 (13.4)	4 (1.6)		
3–4	30 (54.5)	15 (27.3)	10 (18.2)		
Perilesional emphysema, n (%)				26.733 ^c^	<0.001 *
Yes	8 (36.4)	8 (36.4)	6 (27.3)		
No	238 (82.9)	41 (14.3)	8 (2.8)		
Position, n (%)				0.086 ^b^	0.958
Prone	79 (79.8)	16 (16.2)	4 (4)		
Supine	167 (79.5)	33 (15.7)	10 (4.8)		
Lesion morphological characteristics, n (%)				6.598 ^c^	0.112
Solid	221 (80.4)	43 (15.6)	11 (4)		
Ground-glass opacity	8 (66.7)	4 (33.3)	0 (0)		
Cavitary	17 (77.3)	2 (9.1)	3 (13.6)		
Pleural effusion, n (%)				22.568 ^c^	<0.001 *
Yes	7 (41.2)	4 (23.5)	6 (35.3)		
No	239 (81.8)	45 (15.4)	8 (2.7)		
Fissure involvement, n (%)				0.683 ^c^	>0.999
Yes	3 (100)	0 (0)	0 (0)		
No	243 (79.4)	49 (16)	14 (4.6)		
Angle, *n (%)*				9.995 ^b^	0.007 **
≤65	117 (72.7)	34 (21.1)	10 (6.2)		
>65	129 (87.2)	15 (10.1)	4 (2.7)		

* *p* < 0.001; ** *p* < 0.01; *** *p* < 0.05; ^a^ (F), One-Way ANOVA; ^b^ (χ^2^), Pearson Chi-Square test; ^c^ (χ^2^), Fisher’s Exact Test; SD, Standard deviation.

#### 3.3.2. Post-Biopsy Hemorrhage

Post-biopsy hemorrhage was significantly associated with:

- Older age (t = 2.102, *p* = 0.036);
- Smaller lesion diameter (t = 2.084, *p* = 0.038);
- Longer lesion-to-pleura distance (t = 5.804, *p* < 0.001);
- Shorter skin-to-pleura distance (t = 2.738, *p* = 0.007);
- Supine biopsy position (8.6% vs. 1%, *p* = 0.009).

### 3.4. Multivariate Analysis of Risk Factors for Complications

#### 3.4.1. SIR Score Regression Analysis

Multinomial logistic regression (Table 3) demonstrated that:

- SIR1 complications were significantly associated with:
  ○ Smaller lesion diameter (OR = 0.971, 95% CI = 0.946–0.997, *p* = 0.028);
  ○ Presence of pleural effusion (OR = 5.481, 95% CI = 1.278–23.517, *p* = 0.022);
  ○ Needle angle ≤ 65° (OR = 2.585, 95% CI = 1.251–5.343, *p* = 0.010).
- SIR2–4 complications were associated with:
  ○ Decreased skin-to-pleura distance (OR = 0.899, 95% CI = 0.830–0.974, *p* = 0.009);
  ○ Presence of perilesional emphysema (OR = 19.378, 95% CI = 2.626–142.997, *p* = 0.004);
  ○ Presence of pleural effusion (OR = 30.727, 95% CI = 3.962–238.329, *p* = 0.001);
  ○ Needle angle ≤ 65° (OR = 8.125, 95% CI = 1.299–50.814, *p* = 0.025).

**Table 3:** Multinomial Logistic Model Results.

SIR (Reference Group = SIR0)								
Category	Variables	β	SE	Wald	*p*-Value	OR (95% CI)		
SIR1	Intercept	−4.642	1.469	9.981	0.002			
	Age	0.033	0.017	3.833	0.050	1.034 (1.000–1.069)		
	Lesion diameter	−0.029	0.013	4.836	0.028 **	0.971 (0.946–0.997)		
	Skin–pleura distance	−0.016	0.017	0.908	0.341	0.984 (0.952–1.017)		
	Lesion–pleura distance	0.014	0.008	2.984	0.084	1.014 (0.998–1.030)		
	Emphysema high grade	0.859	0.507	2.870	0.090	2.362 (0.874–6.383)		
	Perilesional emphysema	1.149	0.685	2.815	0.093	3.155 (0.824–12.074)		
	Pleural effusion	1.701	0.743	5.242	0.022 **	5.481 (1.278–23.517)		
	Angle ≤ 65°	0.950	0.370	6.574	0.010 **	2.585 (1.251–5.343)		
SIR2–4	Intercept	−7.171	3.181	5.083	0.024			
	Age	0.020	0.035	0.325	0.569	1.020 (0.952–1.093)		
	Lesion diameter	0.036	0.022	2.586	0.108	1.036 (0.992–1.083)		
	Skin–pleura distance	−0.107	0.041	6.844	0.009 *	0.899 (0.830–0.974)		
	Lesion–pleura distance	0.024	0.016	2.394	0.122	1.024 (0.994–1.056)		
	Emphysema high grade	1.571	0.938	2.808	0.094	4.813 (0.766–30.239)		
	Perilesional emphysema	2.964	1.020	8.449	0.004 *	19.378 (2.626–142.997)		
	Pleural effusion	3.425	1.045	10.740	0.001 *	30.727 (3.962–238.329)		
	Angle ≤65°	2.095	0.935	5.016	0.025 **	8.125 (1.299–50.814)		
SIR (Reference Group = SIR1)								
Category	Variables	β	SE	Wald	*p*-value	OR (95% CI)		
SIR2–4	Intercept	−2.530	3.306	0.586	0.444			
	Age	−0.013	0.037	0.132	0.717	0.987 (0.919–1.060)		
	Lesion diameter	0.065	0.024	7.141	0.008 *	1.067 (1.017–1.119)		
	Skin–pleura distance	−0.091	0.041	4.802	0.028 **	0.913 (0.842–0.990)		
	Lesion–pleura distance	0.010	0.015	0.440	0.507	1.010 (0.980–1.041)		
	Emphysema high grade	0.712	0.961	0.548	0.459	2.038 (0.310–13.405)		
	Perilesional emphysema	1.815	1.008	3.240	0.072	6.142 (0.851–44.335)		
	Pleural effusion	1.724	1.042	2.735	0.098	5.606 (0.727–43.243)		
	Angle ≤65°	1.145	0.950	1.455	0.228	3.143 (0.489–20.210)		

* *p* < 0.01; ** *p* < 0.05, β: Regression estimate coefficient; SE, Standard error; CI, Confidence interval; OR, Odds ratio; Number of observations, 309; Likelihood ratio test, LR χ^2^_(16)_ = 96.179; Sig. > χ^2^ ≤ 0.001; Pseudo R^2^ = 0.378.

The comparison of patients with and without post-biopsy hemorrhage is presented in Table 4.

#### 3.4.2. Binary Logistic Regression for Hemorrhage Risk

In the binary logistic regression model (Table 5), independent risk factors for post-biopsy hemorrhage included:

- Smaller lesion diameter (OR = 0.952, 95% CI = 0.908–0.997, *p* = 0.037);
- Increased lesion-to-pleura distance (OR = 1.048, 95% CI = 1.025–1.071, *p* < 0.001);
- Supine patient positioning (OR = 9.031, 95% CI = 1.073–76.029, *p* = 0.043).

**Table 5:** Independent Variables Associated with the Presence of Hemorrhage (Multivariate Logistic Regression Model).

Variables	β	SE	Wald	*p*-Value	OR (95% CI)
Age	0.047	0.030	2.485	0.115	1.048 (0.989–1.111)
Lesion diameter	−0.050	0.024	4.371	0.037 ***	0.952 (0.908–0.997)
Skin–pleura distance	−0.051	0.029	3.156	0.076	0.950 (0.898–1.005)
Lesion–pleura distance	0.047	0.011	16.916	<0.001 *	1.048 (1.025–1.071)
Supine position	2.201	1.087	4.099	0.043 ***	9.031 (1.073–76.029)
Constant	−8.906	3.076	8.381	0.004	0.000

* *p* < 0.001; *** *p* < 0.05; OR, Odds ratio; CI, Confidence interval; Nagelkerke R^2^, 0.365; Model χ^2^, 44.796; *p* < 0.001.

The Nagelkerke R^2^ value was 0.365, indicating that the model explained 36.5% of the variance in hemorrhage occurrence.

## 4. Discussion

This study suggests that, beyond patient- and lesion-related characteristics, operator-modifiable procedural parameters—particularly needle angle and patient positioning—may be associated with complication risk in CT-guided lung biopsy. Our findings indicate that these technical factors may influence the occurrence of pneumothorax and hemorrhage and could represent potentially modifiable procedural considerations for improving procedural safety.

While previous studies have predominantly focused on non-modifiable factors such as emphysema, lesion size, and lesion-to-pleura distance, our results suggest that procedural decisions made by the operator may influence outcomes. This is particularly relevant in clinical practice, where immediate adjustments to technique can be implemented without altering patient selection.

One of the notable findings of this study was the observed association between a needle–pleura angle of ≤65° and major complications (OR = 8.12). However, given the relatively small number of major events, this finding should be interpreted cautiously and requires validation in larger prospective studies. This observation may provide a practical and measurable parameter that can be incorporated into routine procedural planning. A steeper needle trajectory, approaching a perpendicular angle to the pleural surface, likely minimizes pleural disruption and reduces persistent air leakage, thereby lowering the risk of pneumothorax. Although previous studies have suggested an association between needle angle and complication rates, reported thresholds have varied considerably, ranging from 45° to 80° [10,11]. Our findings contribute to the existing literature by defining a clinically applicable cut-off value that may guide safer biopsy techniques.

Similarly, patient positioning emerged as an important determinant of hemorrhage risk. Supine positioning was associated with a significantly higher incidence of post-biopsy hemorrhage, with an approximately nine-fold increase compared to prone positioning. This effect may be explained by gravitational influences on pulmonary perfusion, respiratory motion, and stabilization of the biopsy tract. While patient positioning is often dictated by lesion accessibility, our results suggest that prone positioning, when feasible, may represent a simple and effective strategy to reduce hemorrhage risk.

### 4.1. Pneumothorax and Its Predictors

Pneumothorax was the most common complication in our cohort, occurring in 14.2% of cases, with 1.3% requiring chest tube placement. These findings are consistent with previous reports, in which pneumothorax rates range between 12% and 45%, and chest tube placement is required in approximately 2–15% of cases [3,12]. The primary predictors of pneumothorax identified in our study were increased lesion-to-pleura distance, perilesional emphysema, and a shallow needle trajectory angle.

Lesion-to-pleura distance has been consistently reported as a strong predictor of pneumothorax [5]. Our findings support this observation, demonstrating that deeper lesions are associated with a higher complication risk, likely due to the longer needle path through aerated lung parenchyma, which increases the likelihood of alveolar injury [13]. In particular, lesions located more than 20 mm from the pleura may require careful planning to minimize this risk.

Perilesional emphysema was another significant predictor, with an odds ratio of 19.4 for moderate-to-severe complications. Emphysematous lung tissue is characterized by reduced elasticity and impaired sealing of the needle tract, leading to a higher probability of persistent air leakage [14]. These findings highlight the importance of careful risk stratification in patients with underlying emphysema. In addition, tract sealing techniques such as saline injection have been shown to significantly reduce pneumothorax rates after CT-guided lung biopsy, further reinforcing the concept that pneumothorax is a preventable and modifiable complication [15].

In addition to these established factors, our study suggests that a needle trajectory angle of ≤65° may be associated with increased pneumothorax risk. A more perpendicular approach to the pleura may reduce pleural injury and facilitate faster sealing of the puncture site, thereby limiting air leakage. Adjusting the needle trajectory, when anatomically feasible, may therefore represent a simple and effective strategy to reduce pneumothorax incidence.

### 4.2. Hemorrhage and Its Risk Factors

Post-biopsy hemorrhage occurred in 4.9% of patients, with no cases requiring major intervention. This rate is comparable to previously reported values ranging from 3.6% to 27% [5,16]. Several factors were identified as independent predictors of hemorrhage, including smaller lesion size, increased lesion-to-pleura distance, and supine patient positioning.

Smaller lesions were associated with a higher risk of hemorrhage, likely due to increased technical difficulty and the need for more precise needle manipulation, which may result in greater parenchymal injury [17]. Additionally, deeper lesions were associated with an increased risk of bleeding, consistent with prior studies demonstrating that longer needle paths are more likely to intersect vascular structures [18]. Lesions located near vascular regions or within the lower lobes may therefore require additional caution during planning.

Patient positioning also significantly influenced hemorrhage risk. Supine positioning was associated with higher bleeding rates, which may be related to gravitational effects on pulmonary blood flow and respiratory motion. In contrast, prone positioning may provide greater stability of the biopsy pathway and reduce vascular injury risk. Although positioning is often dictated by lesion accessibility, our findings suggest that it should also be considered as a modifiable risk factor during procedural planning. However, the interpretation of this finding should be made cautiously, as the relatively small number of hemorrhage events in the prone group may have increased the possibility of statistical instability and residual confounding from procedural or lesion-related factors not fully captured in the present analysis.

### 4.3. Clinical Implications

These findings have direct clinical implications for improving the safety of CT-guided lung biopsy. Incorporating operator-modifiable factors into pre-procedural planning may reduce complication rates without compromising diagnostic yield. Specifically, adjusting the needle trajectory to achieve a steeper pleural angle and selecting prone positioning when anatomically feasible may represent practical strategies to minimize pneumothorax and hemorrhage. Additionally, careful evaluation of lesion depth and the presence of emphysema may further assist in risk stratification and procedural optimization.

### 4.4. Comparison with Previous Studies and Future Directions

Our results are consistent with previous studies identifying lesion depth and emphysema as major predictors of pneumothorax, and lesion size and depth as important determinants of hemorrhage risk [5,19]. However, unlike many prior studies, our analysis highlights the significant role of operator-dependent technical factors. The identification of a specific needle angle threshold provides a measurable parameter that may facilitate standardization of biopsy techniques.

In contrast to some reports, we did not observe a significant association between lesion histopathology and complication rates, despite previous findings suggesting increased bleeding risk in cavitary or semi-solid lesions [4,20]. This discrepancy may be related to the relatively small number of non-solid lesions in our cohort. Future studies with larger sample sizes in these subgroups may provide further clarification.

The variability in reported optimal needle angles across studies suggests that additional factors, including operator experience, lung compliance, and lesion location, may influence complication risk. Prospective multicenter studies are needed to validate these findings and establish standardized procedural guidelines. Furthermore, emerging technologies such as cone-beam CT, dual-energy CT, and artificial intelligence-based planning systems may enhance real-time guidance and improve procedural safety [8,13].

## 5. Limitations

This study has several limitations. First, its retrospective design limits the ability to establish causal relationships between identified risk factors and complications. Although significant associations were observed, prospective studies are needed to confirm these findings. Second, this was a single-center study, which may limit the generalizability of the results. Differences in patient populations, CT equipment, operator experience, and institutional biopsy protocols across centers may influence complication rates and procedural outcomes. Therefore, prospective multicenter studies using standardized procedural criteria and real-time data collection are needed to validate the external applicability and reproducibility of these findings across different healthcare settings.

Additionally, the relatively small number of cavitary and ground-glass lesions may have limited the statistical power of subgroup analyses and reduced the reliability of complication risk estimation in these specific lesion subtypes. Because follow-up was primarily limited to the immediate post-procedural period and short-term observation, delayed complications such as late pneumothorax or delayed hemorrhage may have been underdetected, potentially leading to underestimation of the true complication rate.

Although all procedures were performed by experienced interventional radiologists using standardized institutional protocols, inter-operator variability in procedural technique and decision-making may still have influenced complication rates and procedural outcomes. Interobserver variability in angle measurement was not specifically assessed in the present study and may represent an additional source of measurement variability.

Additionally, the relatively small number of major complication events may have limited the stability of the multivariable regression analyses and may have contributed to wide confidence intervals and inflation of some odds ratio estimates. Therefore, these findings should be interpreted cautiously and considered exploratory until validated in larger prospective cohorts.

Furthermore, internal validation methods such as bootstrap resampling or cross-validation were not performed in the present study. Therefore, the reliability and generalizability of the regression models should be interpreted cautiously until validated in larger prospective multicenter cohorts.

In addition, nondiagnostic or insufficient biopsy samples were not systematically analyzed in the present study, as the study cohort primarily consisted of cases with definitive histopathological diagnoses. Therefore, direct assessment of diagnostic yield was beyond the scope of this analysis.

Finally, this study did not include a comparison with alternative biopsy guidance techniques, such as cone-beam CT or ultrasound-guided biopsy, which may have different safety profiles. Comparative studies evaluating these modalities may further refine procedural strategies.

## 6. Conclusions

In conclusion, CT-guided lung biopsy is a safe and effective diagnostic procedure, but it is associated with a risk of complications, most commonly pneumothorax and hemorrhage. Our findings suggest that operator-modifiable factors, particularly needle angle and patient positioning, may influence complication risk during CT-guided lung biopsy. Optimization of these technical parameters may represent a practical strategy for improving procedural safety; however, these findings should be validated in larger prospective studies. Future prospective studies are warranted to validate these findings and to establish standardized guidelines for minimizing complications, improving patient safety and procedural outcomes in routine clinical practice.

## Figures and Tables

**Table 1:** Patient Demographic and Clinical Characteristics.

Variables (*N* = 309)	Descriptive
Age (years), mean (±SD)	64.5 (11.6)
Lesion diameter (mm), mean (±SD)	28.4 (15.2)
Skin–pleura distance (mm), mean (±SD)	40.5 (11.0)
Lesion–pleura distance (mm), mean (±SD)	33.4 (21.3)
Angle, mean (±SD)	65.2 (14.6)
Gender, *n (%)*	
Male	186 (60.2)
Female	123 (39.8)
Lesion location, *n* (%)	
Right upper	73 (23.6)
Right lower	47 (15.2)
Right middle	15 (4.9)
Left upper	91 (29.4)
Left lower	76 (24.6)
Left lingular	7 (2.3)
Lesion histopathology, *n* (%)	
Malign	226 (73.1)
Benign	83 (26.9)
Emphysema grade, *n* (%)	
1–2	254 (82.2)
3–4	55 (17.8)
Perilesional emphysema, *n* (%)	22 (7.1)
Position, *n (%)*	
Prone	99 (32)
Supine	210 (68)
Lesion morphological characteristics, *n* (%)	
Solid	275 (89)
Ground-glass opacity	12 (3.9)
Cavitary	22 (7.1)
Pleural effusion, *n* (%)	17 (5.5)
Fissure involvement, *n* (%)	3 (1)
Complications, *n* (%)	63 (20.4)
Pneumothorax, *n* (%)	44 (14.2)
Hemorrhage, *n* (%)	15 (4.9)
Pneumothorax + hemorrhage, *n* (%)	4 (1.3)

SD, Standard deviation.

**Table 4:** Presence of Hemorrhage According to Certain Patient Characteristics.

Continuous Variables	Hemorrhage (+) (n = 19)	Hemorrhage (−) (n = 290)	t	*p*-Value
Age (years), mean (±SD)	69.8 (7.4)	64.1 (11.7)	2.102 ^a^	0.036 ***
Lesion diameter (mm), mean (±SD)	21.4 (13.5)	28.9 (15.2)	2.084 ^a^	0.038 ***
Skin–pleura distance (mm), mean (±SD)	33.9 (11.3)	41.0 (10.9)	2.738 ^a^	0.007 **
Lesion–pleura distance (mm), mean (±SD)	59.6 (24.8)	31.7 (20.0)	5.804 ^a^	<0.001 *
Angle, mean (±SD)	67.8 (12.2)	65.0 (14.8)	0.796 ^a^	0.427
Ordinal and Nominal Variables	Hemorrhage (+) (n = 19)	Hemorrhage (−) (n = 290)	χ^2^	*p*-Value
Gender, *n (%)*			0.572 ^b^	0.450
Male	13 (7)	173 (93)		
Female	6 (4.9)	117 (95.1)		
Lesion histopathology, n (%)			NA	0.114
Malign	17 (7.5)	209 (92.5)		
Benign	2 (2.4)	81 (97.6)		
Position, *n (%)*			NA	0.009 **
Prone	1 (1)	98 (99)		
Supine	18 (8.6)	192 (91.4)		
Lesion morphological characteristics, n (%)			0.792 ^c^	>0.999
Solid	17 (6.2)	258 (93.8)		
Ground-glass opacity	0 (0)	12 (100)		
Cavitary	2 (9.1)	20 (90.9)		

* *p* < 0.001; ** *p* < 0.01; *** *p* < 0.05; a(t), Independent sample *t*-test; b(χ^2^), Pearson Chi-Square test; SD, Standard deviation; NA: Not applicable.

## Data Availability

The datasets used and/or analysed during the current study are available from the corresponding author on reasonable request.

## Supplementary Materials

The following supporting information can be downloaded at: https://www.mdpi.com/article/10.3390/diagnostics16121792/s1, Table S1: STROBE checklist.
